# Mesenchymal hamartoma of the liver

**DOI:** 10.4103/0971-5851.65338

**Published:** 2009

**Authors:** Rahul Gupta, Sandesh V. Parelkar, Beejal Sanghvi

**Affiliations:** *Department of Pediatric Surgery, King Edward Memorial Hospital, Parel, Mumbai, India*

**Keywords:** *Hemangio-endothelioma*, *mesenchymal hamartoma*, *right hepatectomy*

## Abstract

Mesenchymal hamartoma of the liver is the second most common benign liver tumor in children, yet its biology and pathogenesis are poorly understood. Typically, it presents as a large benign multicystic liver mass in children younger than three years, amenable to complete resection. Most tumors gradually increase in size, some reaching enormous proportions, some can undergo incomplete spontaneous regression, and rarely, few have shown malignant transformation to undifferentiated (embryonal) sarcoma. Here, we report a 13 month-old child who presented with abdominal distension and respiratory distress. Ultrasonography, Computed Tomography (CT), and magnetic resonance imaging (MRI) of the abdomen were suggestive of a mesenchymal hamartoma of the liver. Right hepatectomy was performed. Postoperatively, the patient recovered well. An attempt was also made to understand the possible etiology of the tumor.

## INTRODUCTION

Mesenchymal hamartoma of the liver is a benign tumor of infancy characterized by an admixture of epithelial structures in a loose connective tissue stroma with fluid accumulation suggestive of lymphangiomatous channels. We present a case of mesenchymal hamartoma along with a review of literature, which might be useful in understanding the etiology, pathogenesis, diagnosis, and management of this rare anomaly.

## CASE REPORT

A 13-months-old male child presented with gradual distention of the abdomen over a period of 25 days, with fever, cough, and respiratory distress since four days. On examination; he was afebrile and anicteric. There was visible fullness in the right upper quadrant of the abdomen. The liver was palpable, 5 cm below the right subcostal margin in the mid-clavicular line, non tender and firm in consistency. The superior margin of liver dullness was in the fifth intercostal space. Serum bilirubin was 0.54 mg/dl, SGPT- 22U/L, SGOT- 42U/L, blood urea was 11 mg/dl, Hb - 9.3 gm%, Serum alfa-fetoprotein - 9.81 ng/ml (normal range - 0 to 13.4 ng/ml), and beta Human Chorionic Gonadotrophin - non detectable. Bleeding time, clotting time, prothrombin time, and platelet count were also normal.

An X-ray of the chest showed the elevated right dome of the diaphragm. An ultrasound of the abdomen revealed a large, 10.5 × 9.5 × 10 cm, sized anechoic cystic mass occupying almost the entire right lobe of the liver. There were multiple internal septations within. However, no solid component was visible. The left lobe of the liver was enlarged. The portal vein showed normal flow; its right branch splayed antero-superiorly over the tumor. No portal or retroperitoneal lymphadenopathy was seen nor were there any ascites. A plain and contrast-enhanced CT scan of the abdomen showed a large well-defined cystic mass with few internal septations in the right lobe of the liver, measuring approximately, 10 × 8 × 13 cm, occupying segments V, VI, and VII. There was no calcification, nor was there any soft tissue component within. On a contrast scan, the septae showed enhancement. The mass was seen to displace the middle hepatic vein, the right hepatic vein was not visualized, and the left hepatic vein was normal. The retrohepatic inferior vena cava was compressed. The mass was abutting the portal vein bifurcation. The main portal vein at the porta and its left branch were normal. The anterior branch of the right portal vein was displaced by the mass forming in its anterior margin. The posterior branch of the right portal vein was not seen. The hepatic artery was normal in caliber.

**Figure 1:**
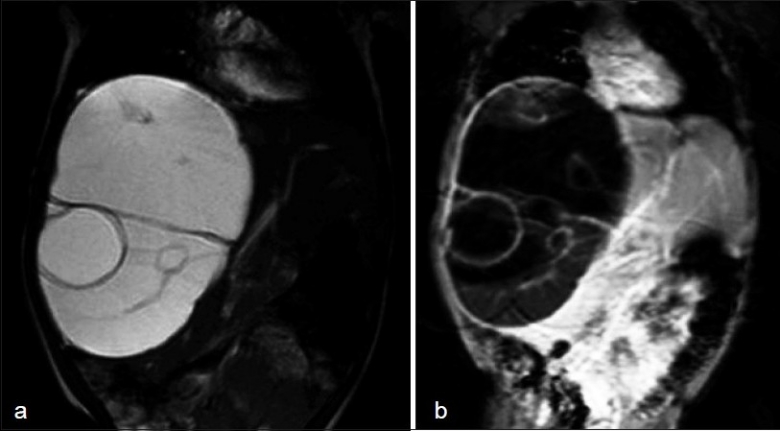
(a) MRI Abdomen (T2 weighted image), (b) MRI Abdomen (T1 weighted image)

On an MRI scan, the lesion was hyper intense on T2W image and hypo intense on T1W images – conforming its cystic nature. Multiple septa were best seen on T2 W images, which demonstrated the complex nature of the cystic mass [Figure 1a and b].

**Figure 2:**
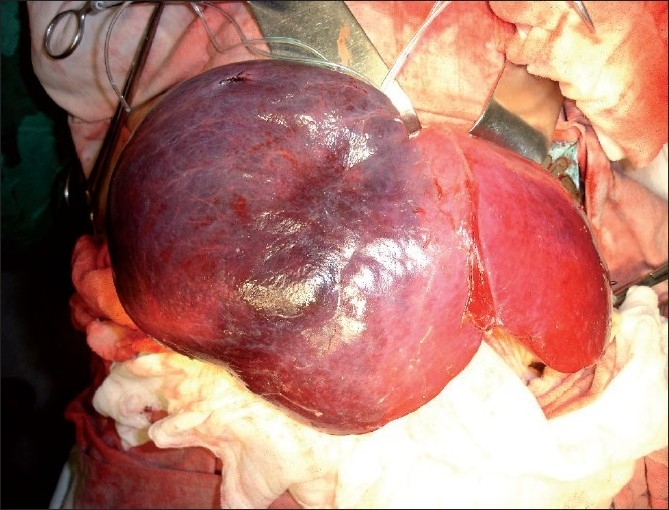
Mesenchymal hamartoma involving Right lobe of liver

The abdomen was explored through a right upper transverse incision and was found to have a mass, 15 × 12 cm, involving segments V, VI, and VII of the right lobe of the liver [Figure 2]. The patient underwent right hepatectomy. The mass was excised completely with a healthy wedge of segment VIII of the liver. Postoperatively, the patient recovered well. On examination, the cut section of the specimen showed areas of multiple, thin-walled, cysts containing straw-colored gelatinous material. On histopathopathological examination, the cysts walls were hyalinized, but no definitive lining was seen. The cyst wall focally showed bile ducts, lymphoid aggregates, and blood vessels. The cysts were separated by a loose mesenchyme, which showed fibromyxoid tissue with inflammation. Cells embedded within it were bile ducts and hepatocytes. On immunohistochemistry, cytokeratin highlighted the disorganized bile duct proliferation and CD 31 and CD 34 highlighted the blood vessels.

## DISCUSSION

Mesenchymal hamartoma is a rare, benign, developmental tumor of the liver, with occasional risk of malignancy. Histologically, it appears as a disordered arrangement of the mesenchyme, bile ducts, and hepatic parenchyma. Cords of normal – appearing hepatocytes are separated by zones of loose, poorly cellular mesenchyme. The porous nature of the mesenchyme permits accumulation of fluid.[1] Grossly, it has stromal and cystic components with no capsules, and can grow to large sizes.[2] Rarely, is the tumor solid.

Although such a mass was first described by Maresh in 1903, Edmondson was the first to use the term “mesenchymal hamartoma” of the liver for describing the four cases that he had observed, and he distinguished mesenchymal hamartoma from other cystic and tumor-like lesions of the liver.[3] He suggested that the lesion might result from a failure in the normal development of the embryonic fetal liver or that some cases might represent a degenerative change of an accessory lobe. Denher *et al*. and Packard and Palmer[4,5] suggested that it was a developmental anomaly of the portal connective tissue during fetal life rather than a true neoplasm. Clinically, patients with mesenchymal hamartoma present in the first two years of life, with a median age of 10 months (0 – 19 years). The male to female ratio is 2:1, the right lobe being affected more frequently than the left (6:1).[1,4]

The typical presentation is one of asymptomatic, rapid abdominal distention with a palpable mass on physical examination. The rapid expansion of the tumor is believed to be due to degeneration of the mesenchyme and fluid accumulation. Other uncommon associated symptoms are vomiting, fever, constipation, diarrhea, and weight loss.[1] Laboratory investigations usually reveal normal liver function with elevated alpha-fetoprotein, which is believed to be secreted by the proliferating hepatocytes within the tumor.[6] The radiological appearance is one of a large, uni - or multicystic, avascular mass occupying part of the liver.[2] Surgical resection has been the standard treatment for this tumor. Marsupialization is also proposed in cases not amenable to resection.[7] In previously reported cases of mesenchymal hamartoma occupying part of the liver, the tumors were successfully treated by surgical resection, and the remaining liver tissue provided adequate liver function.[7] Aspiration of the fluid was undertaken in order to relieve discomfort and reduce tension and to encourage the remaining normal liver tissue to grow. Marsupialization of the cysts was considered if this did not contain the problem.

In summary, mesenchymal hamartoma is one cause of a cystic liver mass; the lesion is benign. Management depends on the location of the lesion and assessment of resectability as depicted by the imaging.
